# Solicitude toward artificial intelligence among health care providers and its relation to their patient’s safety culture in Saudi Arabia

**DOI:** 10.1186/s12913-025-13001-3

**Published:** 2025-07-02

**Authors:** Hamda Ahmed Mohamed Eldesoky, DaifAllah AlThubaity, Abeer Yahia Mahdy Shalby, Fatma Abdelaziz Mohammed

**Affiliations:** 1 Medical-Surgical Nursing Department, Nursing College, Najran University, Najran City, Saudi Arabia; 2 Health Research Center, Najran University, Najran, Saudi Arabia; 3 Nursing Practices Department, Faculty of Nursing, Umm Al-Qura University, Makkah, Saudi Arabia

**Keywords:** Artificial intelligence, Knowledge, Attitudes, Patient safety culture, Healthcare providers

## Abstract

**Background:**

The healthcare sector is undergoing a digital transformation, where the integration of Artificial Intelligence (AI) plays a vital role in reshaping healthcare practices. AI technologies promise to improve work procedures, mitigate future risks, and expedite proactive patient care. This guide seeks to address the significant knowledge gap among healthcare professionals, focusing on AI’s integration within healthcare systems and its impact on patient safety culture.

**Methods:**

A descriptive correlational design was implemented to explore the correlations between healthcare providers’ knowledge and attitudes toward artificial intelligence (AI) and their perception of patient safety culture. A non-probability, convenient sample of 238 healthcare providers comprising physicians, nurses, and technicians working at Najran University Hospital was included in the study. Data collection was conducted using two self-reported questionnaires: the Structured Knowledge and Attitude Questionnaire on Artificial Intelligence and the Hospital Survey on Patient Safety Culture (HSOPSC).

**Results:**

The research findings indicated that the healthcare providers’ knowledge and attitude toward AI are significant predictors of patient safety culture, with a combined explanatory power of 60% (*R*^2^ = 0.60). Both knowledge (B = 0.296, *p* = .000) and attitude (B = 0.502, *p* = .000) show significant positive relationships with patient safety culture, indicating that higher knowledge and a more positive attitude toward AI contribute to a stronger patient safety culture.

**Conclusions:**

This study's findings emphasize the need to enhance healthcare providers’ knowledge and attitudes toward AI to reinforce a culture of patient safety. Healthcare institutions are encouraged to incorporate AI-centered education and training programs to enhance providers’ comprehension and confidence in leveraging AI for safer clinical practice.

**Supplementary Information:**

The online version contains supplementary material available at 10.1186/s12913-025-13001-3.

## Introduction

Saudi Arabia has embarked on a transformative journey to integrate artificial intelligence (AI) into its healthcare system, aligning with the objectives of Vision 2030. This initiative emphasizes enhancing healthcare accessibility, optimizing workflows, and improving crisis management capabilities, as evidenced during the COVID-19 pandemic [1]. The Saudi Data & Artificial Intelligence Authority (SDAIA) has prioritized AI adoption in the healthcare sector through national strategies and investment in digital infrastructure [2].

AI-driven health technologies (AIHTs) include tools that assist in clinical decision-making, diagnostics, treatment planning, and workflow optimization. The successful integration of these technologies depends not only on the available infrastructure but also on healthcare providers’ knowledge and attitudes. *Knowledge* involves understanding AI’s core concepts, benefits, limitations, and ethical concerns, while *attitude* reflects trust, perceived usefulness, concerns over job security, and its impact on provider–patient relationships [3, 4]. Studies show that limited AI literacy among healthcare professionals can hinder effective implementation [5].

The ethical dimension of AI implementation raises the need to preserve humanistic care in clinical settings. The concept of *solicitude* in healthcare refers to a compassionate, ethical, and human-centered orientation that underscores the importance of empathy and moral responsibility in caregiving. It draws from the philosophy of care ethics, emphasizing that healthcare is not merely a technical task but a relational and moral endeavor. In the context of integrating artificial intelligence (AI) into healthcare systems, solicitude serves as a guiding principle that ensures technology augments rather than replaces the human elements of care. While AI offers significant advantages such as increased efficiency, data-driven diagnostics, and predictive analytics it lacks the emotional intelligence and moral reasoning that are integral to human caregiving. Solicitude reminds the healthcare providers that trust, empathy, and the therapeutic relationship between patients and providers are foundational to healing and patient satisfaction. When AI is implemented without regard for these human dimensions, there is a risk of depersonalization, reduced patient engagement, and ethical oversights [6, 7].

Despite the increasing adoption of AI, there is a noticeable lack of research examining how healthcare professionals’ knowledge and attitudes toward AI relate to patient safety culture, particularly in the context of Saudi Arabia, where digital health transformation is a national priority. Existing literature often addresses these concepts independently, without exploring their interplay in real-world practice [8, 9]. Therefore, an important yet underexplored dimension is the relationship between AI adoption and *patient safety culture* which is considered as a shared organizational commitment to minimizing harm through open communication, teamwork, leadership support, and non-punitive error reporting. As a result, how providers perceive and interact with AI may influence their safety behaviors and attitudes toward reporting or addressing system failures [4, 10].

This study addresses this gap by focusing on the healthcare providers who have substantial influence over clinical workflows and patient outcomes. It contributes to the broader understanding of AI readiness, informs policy and training development, and supports ethical, safe implementation of AI technologies in Saudi healthcare [11].

### Theoretical framework

The relationship between healthcare providers’ knowledge and attitudes toward artificial intelligence (AI) and patient safety culture can be better understood through the complementary lenses of the Technology Acceptance Model (TAM) and the Theory of Reasoned Action (TRA). While TAM is rooted in TRA, each model offers distinct yet synergistic insights. TAM emphasizes the importance of perceived usefulness and perceived ease of use as primary drivers of technology adoption. In this context, knowledge about AI such as its clinical applications, limitations, and ethical considerations shapes how useful and manageable providers perceive AI tools to be, which in turn fosters more positive attitudes toward their integration in clinical practice [12].

**Fig. 1:**
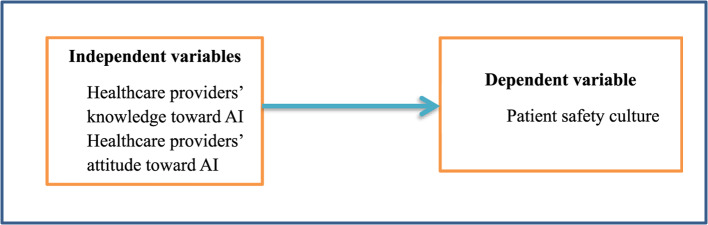
A proposed researchers' study framework

However, TAM primarily focuses on individual cognitive processes and does not fully account for the influence of social context. This is where TRA provides added value. TRA introduces the concept of subjective norms that shape behavioral intentions [13]. Together, these models explain not only why providers form favorable attitudes toward AI but also how those attitudes translate into actual adoption behaviors especially in environments where AI use is encouraged and normalized. In turn, increased AI adoption contributes to a more robust patient safety culture by enhancing decision-making, reducing errors, and optimizing workflows. Thus, targeted training that enhances AI knowledge and fosters a supportive environment can strengthen both individual acceptance and organizational safety practices [8, 14, 15]. Based on that, the researchers hypothesized the following study framework (Fig. 1).

### Artificial intelligence: healthcare providers’ knowledge and attitude

The rapid advancement of artificial intelligence (AI) has transformed various sectors, including healthcare, by enhancing clinical decision-making, improving diagnostic accuracy, and optimizing patient care processes. AI-driven technologies, such as machine learning algorithms, predictive analytics, and robotic-assisted procedures, have the potential to revolutionize healthcare delivery. AI refers to software systems created by humans to accomplish complex tasks. These systems can perceive their environment through data acquisition, interpreting collected data, reasoning based on the knowledge derived from the data, and making decisions on the most appropriate actions to take [16, 17].

However, the effective incorporation of AI into healthcare largely relies on healthcare providers’ knowledge and attitudes toward these technologies. Understanding how healthcare professionals perceive AI, their level of familiarity with its applications, and their willingness to adopt AI-based solutions is crucial for ensuring effective implementation and maximizing patient outcomes. Healthcare providers’ knowledge of AI influences their confidence in applying AI-driven tools, affecting their acceptance as well as utilization of such technologies in clinical settings. A lack of AI-related knowledge may lead to skepticism, resistance, or concerns about ethical and legal implications, thereby hindering adoption [18, 19].

Attitude also has a significant role, as a positive perception of AI’s benefits can encourage its use, whereas fear of job displacement or doubts about AI’s reliability may contribute to reluctance. Many healthcare professionals recognize AI’s capacity to boost diagnostic accuracy, expedite workflows, and enhance patient outcomes, leading to a positive perception of its role in modern medicine. Furthermore, the interplay between AI adoption and patient safety culture is critical, as healthcare providers who understand AI’s role in enhancing safety and efficiency are more likely to integrate it into practice responsibly [19, 20].

### Patient safety culture

Patient safety culture is an essential component of healthcare systems that focuses on minimizing errors, improving patient outcomes, and fostering a safe environment for both patients and healthcare providers. It is defined as the shared values, beliefs, and norms within an organization that influence attitudes and behaviors toward patient safety [21]. The importance of patient safety culture has gained significant attention worldwide, as medical errors and adverse events remain leading challenges in healthcare settings. Establishing a strong patient safety culture requires organizational commitment, open communication, and continuous learning to enhance safety practices and reduce preventable harm [22].

Essential components of patient safety culture encompass leadership support, teamwork, error reporting, staffing levels, and education. Strong leadership fosters safety by setting expectations and ensuring accountability. Effective teamwork and communication reduce errors, particularly in high-risk settings. A non-punitive error reporting system promotes transparency and learning. Adequate staffing prevents burnout and medical errors. Continuous education on safety principles strengthens a safety culture, ensuring sustained improvements in patient care [23–26].

A strong patient safety culture has been linked to improved healthcare outcomes, including decreasing medical errors, lower mortality rates, and higher patient satisfaction. Studies have demonstrated that hospitals with solid safety cultures experience fewer adverse events, as staff members are more vigilant, proactive, and engaged in continuous quality improvement efforts. Additionally, patient-centered care approaches incorporating safety culture principles lead to better communication, increased trust between providers and patients, and improved healthcare experiences [27].

### Significance of the study

Healthcare providers, such as nurses, physicians, and technicians, play distinct yet interdependent roles in delivering high-quality care through a multidisciplinary approach. Nurses, often the primary point of contact for patients, ensure ongoing monitoring, treatment administration, and emotional support. Physicians lead diagnosis and treatment planning, while technicians provide critical diagnostic and technical functions. Artificial intelligence (AI) is increasingly enhancing these roles by supporting clinical decisions, improving diagnostic precision, and reducing administrative and human error burdens. These improvements translate into more efficient workflows and potentially better patient outcomes [28, 29].

Despite these advancements, the successful integration of AI into clinical practice depends heavily on providers’ readiness, particularly their knowledge, attitudes, and the cultural environment within healthcare institutions. Understanding these factors is essential for safe and effective implementation. However, there is limited empirical research assessing the preparedness of healthcare professionals for AI adoption, especially in regions undergoing digital transformation [30].

In Saudi Arabia, healthcare is rapidly evolving under Vision 2030, which includes strategic investments in digital health and artificial intelligence. Yet, few studies have explored how Saudi healthcare providers perceive AI or how prepared they are to use it in ways that uphold patient safety. A recent report revealed that only 39% of Saudi healthcare providers feel confident in their ability to use AI tools, indicating a gap between national strategy and frontline readiness [31].

Therefore, this study aims to address this gap by exploring the relationship between healthcare providers’ knowledge, attitudes toward AI, and patient safety culture. By identifying gaps in training, awareness, and institutional readiness, the findings can guide evidence-based policy development and inform the design of strategic interventions. These may include tailored training programs and targeted awareness campaigns to build a technologically competent and safety-focused workforce. The outcomes will be valuable for healthcare institutions, educators, and policymakers seeking to advance AI integration while maintaining high standards of patient care and safety [2].

### Research questions:

- Q1. What is the level of knowledge of AI technologies among healthcare providers?
- Q2. What is the level of healthcare providers’ attitude toward using artificial intelligence?
- Q3. Is there a relation between healthcare providers’ knowledge and patient safety culture toward using artificial intelligence?
- Q4. Is there a relation between healthcare providers’ attitudes and patient safety culture toward using artificial intelligence?

## Method

### Study design and setting

This study follows a cross-sectional descriptive correlational study to accomplish the research’s goal and answer the research questions. This study was performed at the Najran University Hospital, affiliated with Najran University. This hospital offers comprehensive care across all medical specializations and free services through several departments and units.

### Study participants

The study included 238 healthcare providers comprising physicians, nurses, and technicians working at Najran University Hospital, selected from a total staff of 280. Participants were recruited using a non-probability convenience sampling method based on their accessibility and willingness to take part in the study. Eligibility criteria required participants to have at least three months of continuous employment at the hospital and to provide informed, voluntary consent.

### Study tools

The study implemented two tools to assess the study variables:

#### Tool I: structured knowledge and attitude questionnaire on artificial intelligence

The tool was used to assess healthcare providers’ knowledge and attitudes regarding artificial intelligence (AI) and consists of three main sections:

##### **Section one: demographic and professional characteristics**

This section collects data on participants' demographic and professional attributes, including age, gender, healthcare provider category, years of professional experience, and prior training in AI.

##### Section two: AI knowledge assessment questionnaire

This questionnaire was developed by researchers following an extensive review of relevant literature, including Sur et al., Catalina et al., and Elsayed and Sleem. It consists of 20 multiple-choice questions (MCQs) covering five key domains: (a) benefits of AI, (b) core components and characteristics, (c) roles and strategies, (d) barriers and challenges, and (e) principles and applications. The overall scores for healthcare providers' knowledge of artificial intelligence (AI) were calculated based on responses to 20 MCQs; each correct answer was awarded one point, while incorrect answers received a score of zero. Thus, the total raw score for each participant could range from 0 to 20. To standardize the results, the raw scores were converted into percentage scores using the formula: (raw score/20) × 100. Based on the resulting percent score, knowledge levels were categorized into three groups: a high level of knowledge was defined as a score of 66.6% or higher (equivalent to 14 or more correct answers), a moderate level of knowledge corresponded to scores between 33.3% and less than 66.6% (7 to 13 correct answers), and a low level of knowledge was assigned to those scoring below 33.3% (6 or fewer correct answers) [32–34]. A Cronbach's alpha coefficient is 0.91 revealed high reliability.

##### Section three: general attitudes toward artificial intelligence scale

This scale was developed by Schepman and Rodway to assess the healthcare providers' attitude toward artificial intelligence (AI) using a set of twenty statements, each rated on a five-point Likert scale ranging from 1 ("strongly disagree") to 5 ("strongly agree"). Consequently, the total attitude score for each participant could range from a minimum of 20 to a maximum of 100. To evaluate the overall level of attitude, each total score was converted into a percentage using the formula: (total score/100) × 100. The resulting percent scores were then categorized to determine the respondent's general attitude toward AI. A percent score greater than 60% was interpreted as a positive attitude toward AI, while scores of 60% or below reflected a negative attitude. This scoring method allowed for a standardized interpretation of participants' attitudes across the full range of possible responses [35]. A Cronbach's alpha coefficient is 0.86 revealed high reliability. Please see supplementary No. 1.

#### Tool II: Hospital Survey on Patient Safety Culture (HSOPSC)

The second tool utilized in this research is a structured questionnaire adapted from the Hospital Survey on Patient Safety Culture (HSOPSC) created by the Agency for Healthcare Research and Quality (AHRQ) in 2016. This instrument is intended to evaluate patient safety culture within healthcare settings and comprises 42 statements categorized into 12 dimensions. A reverse score was conducted for the negative statements to ensure consistency in the scoring approach. A five-point Likert scale was used to solicit the participants’ responses, where one indicates (strongly disagree) and five (strongly agree). The overall score ranges from 42 to 210; a mean percent score of greater than 66.6% indicates a higher perception of patient safety culture, while a score between 33.3% to 66.6% reflects a moderate perception. Conversely, a mean percentage score below 33.3% signifies a low perception. The tool exhibited strong reliability with Cronbach's alpha coefficient of 0.88 [36].

### Validity and reliability

The content validity of the developed questionnaire was evaluated by a panel of five academic experts, who were classified as follows: Three professors in medical and surgical nursing, one professor in nursing administration, and one professor in psychiatric and mental health nursing. Based on their feedback, several elements were modified to enhance clarity. The content validity index (CVI) was 0.9, reflecting a high level of agreement among experts regarding the relevance and clarity of the tool items. Also, its reliability was assessed using Cronbach's alpha correlation coefficient to determine internal consistency. The findings demonstrated excellent internal consistency, as evidenced by Cronbach’s alpha coefficient of 0.91.

A pilot study was conducted on 5% (*n* = 12) of the target population to assess the instruments’ clarity, feasibility, and applicability and estimate the time required for completion. The pilot study's findings indicated that the tools were comprehensible and appropriate for the study population. Consequently, no modifications were made to the final versions of the instruments.

### Data collection procedure

Data collection was conducted over three months, from March to May 2024. The study utilized an online questionnaire designed and administered through Google Forms to ensure accessibility and efficiency in data gathering. To facilitate participant recruitment, the researchers developed a concise one-page informational document disseminated to study participants via social media platforms, including WhatsApp and e-mail. This document provided comprehensive details about the study, including its objective, methodology, voluntary participation, confidentiality measures, and questionnaire completion guidelines. All questionnaire items were carefully designed to ensure completeness and comprehensiveness, enhancing the collected data's reliability and validity.

### Ethical approval

The research proposal obtained official approval (No. 202402–076–018368–041481) from the Ethics Committee at Najran University. Following this, an official authorization letter was obtained from the College of Nursing at Najran University, outlining the purpose of the study. This letter was subsequently submitted to the relevant authorities at Najran University Hospital to secure permission for data collection. The study was conducted following the Declaration of Helsinki. Before participation, informed consent was obtained from all study participants. The electronic questionnaire commenced with a declaration of informed consent, which participants acknowledged before proceeding. This declaration emphasized the voluntary nature of participation, the right to withdraw at any time without consequences, and the confidentiality and anonymity of the data collected. Participants were explicitly informed that the data would be used exclusively for research purposes and that their privacy would be safeguarded throughout the study.

### Data analysis

The reliability of the study instruments was assessed using Cronbach’s Alpha coefficient to determine internal consistency. Descriptive statistics, including frequency and percentage, were used to analyze participants’ demographic characteristics. A mean percent score was utilized to classify participants’ perceptions regarding patient safety culture, the level of healthcare providers’ knowledge, and attitude regarding AI. To explore the relationship between healthcare providers’ knowledge and attitudes regarding AI and patient safety culture, Pearson's correlation coefficient was used to assess the strength and direction of associations. Additionally, multiple regression analysis was conducted to examine the predictive effect of AI knowledge and attitude on patient safety culture. The regression model estimated the unstandardized (B) and standardized (β) coefficients, 95% confidence intervals (CIs), and p-values to determine statistical significance. A *p*-value < 0.05 was considered statistically significant, indicating a meaningful predictive relationship between the independent and dependent variables.

## Results

Table 1 reveals the distribution of healthcare providers according to their demographic characteristics. More than one-third (41.6%) are in the age group of 34 to less than 45, with an average age of 37.18 ± 8.124 years. 63.4% were female, and 52.1% were staff nurses. Most participants (43.7%) have 5 to less than 10 years of experience, with an overall average of 7.16 ± 2.85 years. A significant portion (59.2%) have attended training courses on artificial intelligence (AI), indicating a growing interest in AI applications in healthcare. However, 40.8% have not received such training.

**Table 1:** Distribution of healthcare providers according to their demographic & professional data

**Demographic & professional data**	**No. (*n* = 238)**	**%**
Age (Years)		
< 25	24	10.1
25 < 34	60	25.2
34 < 45	99	41.6
45 +	55	23.1
Minimum – Maximum (years)	22–54	
Mean ± SD	37.18 ± 8.124	
Gender		
Male	87	36.6
Female	151	63.4
Healthcare provider category		
Staff Nurse	124	52.1
Physician	90	37.8
Technician	24	10.1
Years of experience		
< 5	80	33.6
5 < 10	104	43.7
10 < 15	54	22.7
Mean ± SD	7.16 ± 2.85	
Prior training in AI		
Yes	141	59.2
No	97	40.8

Table 2 reflects the healthcare providers’ knowledge and attitudes toward AI in healthcare. This table shows a moderate mean percent score of overall knowledge of AI-related topics (63.46 ± 9.48%). The highest mean knowledge percent score was observed in the benefits and importance of AI (72.40 ± 22.70%), indicating a strong awareness of AI’s potential impact. Principles and applications (64.84 ± 17.36%) and roles and strategies (61.44 ± 15.49%) also scored relatively well. However, knowledge of core components and characteristics (58.96 ± 21.02%) and problems and barriers (60.08 ± 21.86%) is the lowest mean percent score. Concerning the healthcare providers’ attitudes, they held a positive but moderate attitude toward AI (60.59 ± 8.54%).

**Table 2:** Descriptive statistics of healthcare providers’ knowledge and attitude toward Artificial Intelligence (AI)

**Healthcare providers knowledge and attitude toward AI**	**Minimum % score**	**Maximum % score**	**Mean % score**
Benefits and Importance	33.3	100.0	72.40 ± 22.70
Core Components and Characteristics	0.0	100.0	58.96 ± 21.02
Role and strategies	25.0	100.0	61.44 ± 15.49
Problem and Barriers	0.0	100.0	60.08 ± 21.86
Principles and Applications	16.67	100.0	64.84 ± 17.36
Overall Healthcare providers’ AI knowledge	35.0	80.0	63.46 ± 9.48
Overall Healthcare providers’ attitudes toward AI	36.00	82.00	60.59 ± 8.54

Table 3 declares moderate perceptions of patient safety culture among healthcare providers, with a composite mean score of 54.89 ± 8.64%. The highest-rated dimension was hospital management support for patient safety (62.42 ± 23.45%). Other higher-scoring areas included manager expectations and actions promoting patient safety (57.92 ± 19.91%), feedback and communication about error (57.42 ± 17.35%), and teamwork within units (57.01 ± 17.55%). However, communication openness (51.03 ± 16.16%), non-punitive response to error (51.06 ± 19.54%), and frequency of event reporting (51.40 ± 17.79%) scored lower.

**Table 3:** Descriptive statistics of patient safety culture among healthcare providers

**Patient safety culture dimensions**	**Minimum % score**	**Maximum % score**	**Mean % ± SD**
1. Overall perceptions of patient safety	20.0	100.0	52.81 ± 20.69
2. Frequency of event reporting	20.0	93.33	51.40 ± 17.79
3. Teamwork within units	20.00	90.00	57.01 ± 17.55
4. Organizational learning continuous improvement	26.67	86.67	54.62 ± 15.97
5. Hospital handoffs and transitions	25.0	95.0	54.09 ± 18.36
6. Communication openness	26.67	93.33	51.03 ± 16.16
7. Staffing	20.0	90.00	53.10 ± 18.35
8. Non-punitive response to error	20.0	93.33	51.06 ± 19.54
9. Feedback and communication about error	20.0	93.33	57.42 ± 17.35
10. Teamwork across hospital units	20.0	100.00	55.58 ± 22.27
11. Manager expectations and actions promoting patient safety	25.00	100.00	57.92 ± 19.91
12. Hospital management support for patient safety	20.00	100.00	62.42 ± 23.45
Overall patient safety culture	29.52	73.81	54.89 ± 8.64

Table 4 reveals the correlation matrix of the studied variables. There was a strong positive relationship between healthcare providers' AI knowledge and patient safety culture (*r* = 0.695, *p* = 0.000). Additionally, there was a highly statistically significant correlation between healthcare providers' attitude toward AI and patient safety culture (*r* = 0.739, *p* = 0.000).

**Table 4:** Correlation matrix between knowledge, attitude of the healthcare providers toward AI, and patient safety culture

**Variable (% score)**		**Overall patient safety culture**
Overall knowledge	r	.695^**^
	Sig. (2-tailed)	.000
Overall attitude	r	.739^**^
	Sig. (2-tailed)	.000

Pearson Correlation(r)

^**^Correlation is significant at the 0.01 level (2-tailed)

Table 5 shows that the multiple linear regression model demonstrates that knowledge and attitude toward AI are significant predictors of patient safety culture, with a combined explanatory power of 60% (*R*^2^ = 0.60). Both knowledge (B = 0.296, *p* = 0.000) and attitude (B = 0.502, *p* = 0.000) show significant positive relationships with patient safety culture, indicating that higher knowledge and a more positive attitude toward AI contribute to a stronger patient safety culture. Among the two predictors, attitude (Beta = 0.496) has a stronger influence on patient safety culture than knowledge (Beta = 0.325), suggesting that healthcare providers' perceptions and openness to AI play a more critical role in shaping patient safety culture. The significant F-test (F = 170.914, *p* = 0.000) confirms the model is a good fit for predicting patient safety culture.

**Table 5:** The best-fitting multiple linear regression model for the total knowledge, attitude, and patient safety culture score

**Variables**	**Unstandardized coefficients**		**Standardized coefficients**	**t-test**	***p*-value**	**95.0% confidence interval for B**	
	**B**	**Std. Error**	**Beta**			**Lower bound**	**Upper bound**
(Constant)	5.635	2.688		2.096	.037	.338	10.931
Knowledge	0.296	.057	.325	5.202	.000	.184	.409
Attitude	0.502	.063	.496	7.942	.000	.378	.627

Model fit indices

*R*^2^ = 0.60

Model ANOVA: F = 170.914, *P* = 000*

## Discussion

The integration of artificial intelligence (AI) into healthcare transforms clinical decision-making, patient monitoring, and administrative tasks, contributing to an enhanced patient safety culture. AI-driven technologies such as diagnostic algorithms and predictive analytics can improve efficiency, minimize medical errors, and promote better patient outcomes. However, successful AI adoption depends on healthcare providers' knowledge and attitudes toward AI [29, 37].

Based on that, this study found that healthcare providers at Najran University Hospital demonstrated moderate knowledge and moderately positive attitudes toward artificial intelligence (AI), with 40.8% reporting no prior AI-related training. Despite limited formal education, many providers expressed openness toward AI, suggesting that perceived usefulness, central to the Technology Acceptance Model (TAM), may play a stronger role than actual knowledge in influencing acceptance. The moderate attitude levels also align with the Theory of Reasoned Action (TRA), which posits that behavior is shaped by attitudes and social norms; concerns about AI replacing human roles or disrupting patient relationships likely contribute to these cautious attitudes.

These findings highlight the need for structured AI education that enhances both technical understanding and trust. The gap between exposure to AI and the lack of training creates potential liability and patient safety risks, reinforcing the importance of institutional support. Linking TAM and TRA to these results suggests that boosting perceived usefulness, addressing ethical concerns, and fostering a supportive culture can improve AI adoption while ensuring safe and responsible implementation in clinical practice.

These results align with earlier findings by Topol and Rajkumar et al. [38], who noted that AI literacy is often limited due to gaps in training. Also, the study of Ahmed et al. and Gulati et al. emphasizes the value of structured educational initiatives in improving knowledge and acceptance [28, 38–40]. However, recent work by Sommer et al. highlights persistent knowledge gaps, particularly among nurses [41].

Regarding patient safety culture, providers reported a moderate perception, potentially due to ongoing challenges in communication, staffing, and non-punitive error reporting. Similar findings were documented by Hadad et al., Berry et al., Han et al., Skoogh et al., Alrabae et al., and Alquwez et al., who reported a moderate level and emphasized the importance of strong teamwork, communication, and management support in patient safety. Otherwise, Muftawu et al. reported even lower levels of patient safety culture [42–48].

Importantly, this study demonstrated that knowledge and attitudes toward AI significantly predict patient safety culture (*R*^2^ = 0.60, *p* < 0.001), with attitude showing a stronger influence, emphasizing positive perceptions and understanding of AI, enhancing its potential to improve safety outcomes and reduce errors in healthcare settings. Consequently, the findings of this study provide valuable insights into the relationship between healthcare providers' knowledge and attitudes toward artificial intelligence (AI) and patient safety. This is supported by Gulati et al. [40] and Zhang et al. [49], who found that positive attitudes toward AI improved diagnostic accuracy and team collaboration [41, 49]. Also, Rajkomar et al. (2019) and Topol highlighted that healthcare professionals with higher AI literacy demonstrate better integration of AI tools into clinical practice and improve patient outcomes [28, 38]. However, Mesko et al. and Morley et al. argue that knowledge alone cannot overcome systemic issues such as regulatory gaps or ethical concerns [50, 51].

Recent studies conducted in Saudi Arabia provide valuable insights into healthcare providers' evolving knowledge and attitudes toward artificial intelligence (AI) and its implications for patient safety culture. Serbaya et al. [52] and Alsharif et al. [53, 54] found that healthcare professionals generally exhibit mild to moderately positive attitudes toward AI, though concerns about job displacement, error liability, and ethical issues persist. These attitudes were significantly influenced by exposure to AI in clinical settings and prior training. Alshammari et al. [55, 56] reported a moderate awareness of patient safety culture among healthcare providers, highlighting barriers such as communication gaps and medication safety challenges. Meanwhile, studies by Rahman and Qattan [57], Alhamid [58], and Saeed et al. [59] emphasized Saudi Arabia's growing investment in AI technologies under Vision 2030, noting improved operational efficiency and diagnostic accuracy as key benefits. However, they also stressed the need for structured education, supportive infrastructure, and ethical frameworks to ensure effective AI integration and enhance patient safety outcomes [52, 59].

## Conclusion

This study demonstrates that healthcare providers' knowledge and attitude toward AI are significant predictors of patient safety culture, with attitude exerting a stronger influence. These findings highlight the critical role of perception and openness toward AI in creating a safer healthcare environment. The results also confirm the model's validity, reinforcing the importance of targeted interventions that enhance AI literacy and promote positive attitudes among healthcare professionals. Importantly, this study offers a unique contribution by contextualizing these insights within the Saudi healthcare system. It underscores the urgent need to incorporate AI-focused training and mindset-shifting initiatives into national workforce development strategies aligned with Vision 2030. By addressing educational gaps and attitudinal barriers, Saudi healthcare policymakers and institutions can better harness AI's potential to enhance patient safety and drive sustainable improvements in healthcare quality.

### Implications

This study highlights the necessity of enhancing healthcare providers' knowledge and attitudes toward AI to strengthen patient safety culture. It is recommended that healthcare institutions integrate AI-focused education and training programs to improve providers' understanding and confidence in utilizing AI for safer clinical practice. Additionally, fostering a positive attitude through awareness campaigns and hands-on AI workshops can address misconceptions and encourage greater acceptance of AI technologies. Hospital administrators and policymakers should develop clear guidelines for AI adoption, emphasizing its role in improving patient safety outcomes.

Furthermore, healthcare organizations should focus on seamless AI integration into clinical workflows to enhance decision-making and minimize errors. The study's implications suggest that a positive perception of AI has a greater impact on patient safety culture than knowledge alone, underscoring the need for strategic interventions that go beyond technical training to address attitudinal barriers. These findings provide a foundation for future research on AI-driven innovations in healthcare quality and risk management, ensuring that technological advancements contribute effectively to patient safety culture.

### Limitations of the study

This study has specific limitations that must be acknowledged when interpreting the results. First, the study was performed within a specific healthcare setting, which may restrict the generalizability of the findings to other institutions or healthcare systems with varying infrastructures and levels of AI integration. Second, a cross-sectional design captures data at a single point in time, preventing the assessment of causal relationships between AI knowledge, attitude, and patient safety culture. Additionally, self-reported measures may be subject to social desirability bias, as participants might have provided responses they perceived as favorable rather than reflecting their true knowledge and attitudes. Furthermore, the study did not account for other potential influencing factors, such as organizational readiness, availability of AI resources, or prior exposure to AI technologies, which may have impacted the findings. Future research should address these limitations by incorporating longitudinal designs, diverse healthcare settings, and objective measures of AI competency and patient safety outcomes.

## Supplementary Information

Supplementary Material 1.

## Data Availability

The datasets used and/or analyzed during the current study are available from the corresponding author upon reasonable request.

## Abbreviations

AI: Artificial Intelligence
SDAIA: The Saudi Data & Artificial Intelligence Authority
AIHTs: AI-driven health technologies
TAM: Technology Acceptance Model
TRA: Theory of Reasoned Action.
HSOPSC: Hospital Survey on Patient Safety Culture
