# Supplementary material for: Solicitude toward artificial intelligence among health care providers and its relation to their patient’s safety culture in Saudi Arabia
Source: BMC Health Serv Res. 2025 Jul 2;25:889. doi: 10.1186/s12913-025-13001-3 (PMC12224498; doi:10.1186/s12913-025-13001-3)
Supplement: Supplementary file 1 — Supplementary Material 1. [file 12913_2025_13001_MOESM1_ESM.docx]

***Supplement NO 1***

**Tool I: Structured Knowledge and Attitude Questionnaire on Artificial Intelligence:** The tool was designed to assess healthcare providers' knowledge and attitudes regarding artificial intelligence (AI).

It consists of three main sections :

1. **Demographic and Professional Characteristics:**

This section collects data on participants' demographic and professional attributes, including age, gender, healthcare provider category, years of professional experience, and prior training in AI.

1. **AI Knowledge Assessment Tool:**

This component consists of 20 multiple-choice questions (MCQs) covering five key domains: (a) benefits of AI, (b) core components and characteristics, (c) roles and strategies, (d) barriers and challenges, and (e) principles and applications. Each correct response is assigned a score of 1, whereas incorrect answers receive 0. The scores ranged from 0 -20; each set of questions had their total score converted into a percent score. Total knowledge score was classified by using the following scoring system: a high level of knowledge if the percent score was 66.6% or more, the scores between 33.3% to 66.6% reflected a moderate level, and a low knowledge of AI if less than 33.3%.

1. **General Attitudes Toward Artificial Intelligence Scale:**

This section comprises twenty statements measured using a five-point Likert scale, ranging from 1 "strongly disagree" to 5 "strongly agree." The overall attitude scale goes from 20 to 100, and the percent was categorized as follows: a positive attitude toward AI greater than 60%, and a lower score indicates a negative attitude.

**Section 1:** **Demographic and Professional Characteristics.**

**1. Age (years):**

**2. Gender:** 1. Male ( ) 2. Female ( )

**3. Marital status:** 1. Single ( ) 2.Married ( ) 3.Divorced ( ) 4.Widow ( )

**4. Job category:** 1**.** Staff nurse ( ) 2. Physician ( ) 3.Technician ( )

**5. Years of experience: (……..years)**

**6. Have you attended any previous training regarding AI?** 1.yes ( ) 2.No ( )

**Section 2: AI Knowledge Assessment Tool:**

***Benefits and Importance***

1. **What is one of the primary benefits of AI in healthcare?**
   1. AI replaces the need for healthcare professionals.
   2. AI improves the accuracy of diagnoses and treatment plans.
   3. AI reduces the need for medical research.
   4. AI eliminates patient interactions with healthcare providers.

**Answer: (B)** AI improves the accuracy of diagnoses and treatment plans.

1. **In what way does AI enhance the efficiency of healthcare systems?**
2. AI reduces the need for patient data management.
3. AI eliminates the need for healthcare workers.
4. AI prevents the collection of patient data.
5. AI automates administrative tasks, like scheduling and billing, to save time.

**Answer: (D)** AI automates administrative tasks, like scheduling and billing, to save time.

1. **How can AI help improve the quality of care in healthcare settings?**
   1. By assisting healthcare providers in delivering evidence-based treatments and preventing errors.
   2. By reducing the number of healthcare workers involved in patient care.
   3. By replacing healthcare providers' roles entirely.
   4. By minimizing patient interaction with healthcare providers.

**Answer: (A)** By assisting healthcare providers in delivering evidence-based treatments and preventing errors.

**Core Components and Characteristics**

### **What is a core component of AI that enables machines to learn from data?**

- 1. Blockchain.
  2. Manual data entry.
  3. Traditional programming.
  4. Machine Learning.

**Answer: (D)** Machine Learning.

1. **What is a major characteristic of AI-driven predictive analytics in healthcare?**
   1. AI can predict disease risks based on patient data.
   2. AI prevents early disease detection.
   3. AI ignores past patient records.
   4. AI eliminates the need for medical tests.

**Answer: (A)** AI can predict disease risks based on patient data.

1. **Which of the following is a core component of AI used for natural language processing (NLP) in healthcare?**
   1. Manual documentation only.
   2. Speech recognition and text analysis.
   3. Traditional patient charts.
   4. Paper-based transcription.

**Answer: (B)** Speech recognition and text analysis.

**Role and strategies**

1. **What is one of the primary roles of AI in healthcare?**
   1. Replacing doctors completely in clinical decision-making.
   2. Eliminating the need for patient data in healthcare.
   3. Slowing down medical research to prevent errors.
   4. Assisting in diagnosis and treatment recommendations.

**Answer: (D)** Assisting in diagnosis and treatment recommendations

1. **How does AI contribute to medical imaging and diagnostics?**
   1. By analyzing medical images and detecting abnormalities faster and more accurately.
   2. By manually reviewing all X-rays and MRIs.
   3. By replacing radiologists in hospitals.
   4. By slowing down the diagnostic process ensures accuracy.

**Answer: (A)** By Analyzing medical images and detecting abnormalities faster and more accurately.

1. **Which strategy can help overcome barriers to AI implementation in healthcare?**
   1. Avoiding AI integration in hospitals.
   2. Ignoring regulatory and ethical concerns.
   3. Providing AI training programs for healthcare professionals.
   4. Using outdated AI models to save costs.

**Answer: (C)** Providing AI training programs for healthcare professionals.

1. **How can healthcare systems support AI implementation?**
   1. Establishing policies that balance innovation with patient safety.
   2. Removing all regulations to encourage AI adoption.
   3. Preventing AI from being used in hospitals.
   4. Ignoring AI-related legal and ethical issues.

**Answer: (A)** Establishing policies that balance innovation with patient safety.

**Problem and Barriers**

1. **Why do healthcare professionals sometimes resist AI implementation?**
   1. AI provides 100% accurate diagnoses.
   2. Fear of job displacement and lack of trust in AI decisions.
   3. AI is easy to use and requires minimal training.
   4. AI can replace all healthcare roles without issues.

**Answer: (B)** Fear of job displacement and lack of trust in AI decisions

1. **Which of the following is a major barrier to AI adoption in healthcare?**
   1. Unlimited availability of patient data.
   2. Full acceptance by healthcare professionals.
   3. Complete elimination of medical errors.
   4. High cost of implementation.

**Answer: (D)** High cost of implementation.

1. **A significant limitation of AI in healthcare decision-making is:**
   1. AI never makes errors.
   2. AI lacks human intuition and empathy.
   3. AI completely replaces the need for doctors.
   4. AI can function without patient data.

**Answer:** b) AI lacks human intuition and empathy.

1. **Which of the following is a primary ethical concern regarding AI in healthcare?**
   1. AI systems are too cheap to implement.
   2. AI decision-making is always transparent and easy to understand.
   3. AI eliminates the need for doctors and nurses.
   4. AI can lead to biased treatment outcomes due to biased training data.

**Answer: (D)** AI can lead to biased treatment outcomes due to biased training data.

**Principles and Applications**

1. **Which of the following is a fundamental principle of AI in healthcare?**
   1. AI should replace all healthcare professionals.
   2. AI should enhance, not replace, human decision-making.
   3. AI should function without ethical considerations.
   4. AI should work without patient data.

**Answer:** b) AI should enhance, not replace, human decision-making.

1. **What principle should AI follow to ensure ethical healthcare implementation?**
   1. AI should prioritize automation over human involvement.
   2. AI should operate without regulatory approval.
   3. AI should function without patient consent.
   4. AI should maintain patient privacy and data security.

**Answer: (D)** AI should maintain patient privacy and data security.

1. **One of the major applications of AI in healthcare is:**
   1. Diagnosing and predicting diseases using machine learning algorithms.
   2. Eliminating the need for hospitals and clinics.
   3. Reducing the number of healthcare workers.
   4. Preventing doctors from making treatment decisions.

**Answer:** a) Diagnosing and predicting diseases using machine learning algorithms.

1. **Which AI technology is commonly used for virtual health assistants?**
   1. Blockchain.
   2. Chatbots and Natural Language Processing (NLP).
   3. Manual record-keeping.
   4. Paper-based medical reports.

**Answer: (B)** Chatbots and Natural Language Processing (NLP).

1. **Which of the following is NOT a current AI application in healthcare?**
2. AI-powered robotic surgery
3. AI-generated patient holograms for virtual treatment
4. AI-driven disease prediction models
5. AI-enhanced medical image analysis

**Answer: (B)** AI-generated patient holograms for virtual treatment

1. **What AI-driven technology is used for remote patient monitoring?**
   1. Wearable devices and smart sensors.
   2. Traditional paper-based patient records.
   3. In-person doctor visits only.
   4. AI-powered manual diagnosis.

**Answer: (A)** Wearable devices and smart sensors

**Section 3: General Attitudes Toward Artificial Intelligence Scale**

This section comprises twenty statements measured using a five-point Likert scale, ranging from 1 "strongly disagree" to 5 "strongly agree." Please complete the following scale, indicating your response to each item. There are no right or wrong answers. We are interested in your personal views.

| **Items** | **Strongly Disagree**  **(1)** | **Disagree**  **(2)** | **Neutra**  **(3)** | **Agree**  **(4)** | **Strongly agree**  **(5)** |
| --- | --- | --- | --- | --- | --- |
| 1. I am interested in using artificially intelligent systems |  |  |  |  |  |
| 2. There are many beneficial applications of AI |  |  |  |  |  |
| 3. AI is exciting |  |  |  |  |  |
| 4. AI can provide new economic opportunities for My organization |  |  |  |  |  |
| 5. I would like to use AI in my own job |  |  |  |  |  |
| 6. An AI agent would be better than an employee in many routine jobs |  |  |  |  |  |
| 7. I am impressed by what AI can do |  |  |  |  |  |
| 8. AI can have positive impacts on Nurses' wellbeing |  |  |  |  |  |
| 9. AI systems can help Nurses feel happier |  |  |  |  |  |
| 10. AI systems can perform better than humans |  |  |  |  |  |
| 11. Much of organizations will benefit from a future full of AI |  |  |  |  |  |
| 12.For routine transactions, I would rather interact with an AI system than with a human |  |  |  |  |  |
| 13. I think Artificial Intelligence is dangerous |  |  |  |  |  |
| 14. Organizations use AI unethically |  |  |  |  |  |
| 15. I find AI sinister |  |  |  |  |  |
| 16. AI is used to spy on Nurses |  |  |  |  |  |
| 17. I shiver with discomfort when I think about future uses of AI |  |  |  |  |  |
| 18. AI might take control of healthcare providers |  |  |  |  |  |
| 19. I think AI systems make many errors |  |  |  |  |  |
| 20. Healthcare providers like me will suffer if AI is used more and more |  |  |  |  |  |
